# Puerperal Convulsions

**Published:** 1882-02

**Authors:** Henry Gibbons


### PUERPERAL CONVULSIONS.

Several cases in which pilocarpin, by mouth and hypodermically, was used in eclampsia, are reported with varying results. Langer asserts that it excites uterine contractions and renders them more powerful, and, in two or three cases, as many physicians report a similar result; but Kroner used (*Amer. Jour. Obstet*) injections of pilocarpin in four cases without any appreciable effect upon the uterus, although the toxic effect of the drug was marked.

The weight of opinion seems to favor chloral in large doses by the rectum. Guyot (France) reports remarkable success, thirteen of fourteen cases being saved. He injected into the rectum from one to four drachms in twenty-four hours. Dr. Goodell believes it the best single remedy. He directs a drachm by rectum, or twenty grains by mouth, repeated as often as may be necessary, and asserts that he has never lost a case. Other writers are equally laudatory of chloral, while none discard chloroform. With regard to the induction of premature labor in eclampsia, there seems to be a growing sentiment in its favor, and successful cases are recorded.

Blood-letting is apparently growing in favor again. Many writers advocate it, or at least speak of it as a too much neglected remedy. Dr. C. C. P. Clark (*Am. Jour. Obstet.*) is a strong advocate for the use of morphia in heroic doses. He argues that a woman who bears her pregnancy lightly never has convulsions, hence a prophylaxis consists in removing all irritating conditions. In eclampsia the nervous system is peculiarly tolerant of opiates. Ordinary doses are useless. Inject at once into the arm a *grain and a half of morphia*; should the paroxysm return any time after two hours, repeat the dose. If in labor, repeat the dose in eight hours. He says: "This quantity may look large, but I am perfectly confident, after having tried it many times, that it is perfectly safe. I am almost prepared to swear that twice the quantity, not repeated, would do no harm to a patient in a strongly eclamptic condition."—Dr. Henry Gibbons, Jr., *Pacific Medical and Surgical Journal.*

